# Timing Uncertainty in Collective Risk Dilemmas Encourages Group Reciprocation and Polarization

**DOI:** 10.1016/j.isci.2020.101752

**Published:** 2020-10-31

**Authors:** Elias Fernández Domingos, Jelena Grujić, Juan C. Burguillo, Georg Kirchsteiger, Francisco C. Santos, Tom Lenaerts

**Affiliations:** 1 AI lab, Computer Science Department, Vrije Universiteit Brussel, Pleinlaan 9, 3rd Floor, 1050 Brussels, Belgium; 2 MLG, Département D’Informatique, Université Libre de Bruxelles, Boulevard Du Triomphe, CP 212, 1050 Brussels, Belgium; 3 Department of Telematic Engineering, University of Vigo, 36310 Vigo, Spain; 4 ECARES, Université Libre de Bruxelles, Av. Roosevelt 42, CP 114, 1050 Brussels, Belgium; 5 INESC-ID and Instituto Superior Técnico, Universidade de Lisboa, IST-Taguspark, 2744-016 Porto Salvo, Portugal; 6 ATP-group, 2744-016 Porto Salvo, Portugal

**Keywords:** Behavioral Neuroscience, Data Analysis, Decision Science

## Abstract

Social dilemmas are often shaped by actions involving uncertain returns only achievable in the future, such as climate action or voluntary vaccination. In this context, uncertainty may produce non-trivial effects. Here, we assess experimentally — through a collective risk dilemma — the effect of timing uncertainty, i.e. how uncertainty about when a target needs to be reached affects the participants' behaviors. We show that timing uncertainty prompts not only early generosity but also polarized outcomes, where participants' total contributions are distributed unevenly. Furthermore, analyzing participants' behavior under timing uncertainty reveals an increase in reciprocal strategies. A data-driven game-theoretical model captures the self-organizing dynamics underpinning these behavioral patterns. Timing uncertainty thus casts a shadow on the future that leads participants to respond early, whereas reciprocal strategies appear to be important for group success. Yet, the same uncertainty also leads to inequity and polarization, requiring the inclusion of new incentives handling these societal issues.

## Highlights

• Timing uncertainty influences experimental observations in the collective risk game
• It induces subjects to contribute earlier and in a polarized manner
• Successful players adopt reciprocal strategies, responding in kind to past actions
• Coordination gets more difficult under high timing uncertainty

## Graphical Abstract

**Figure:**
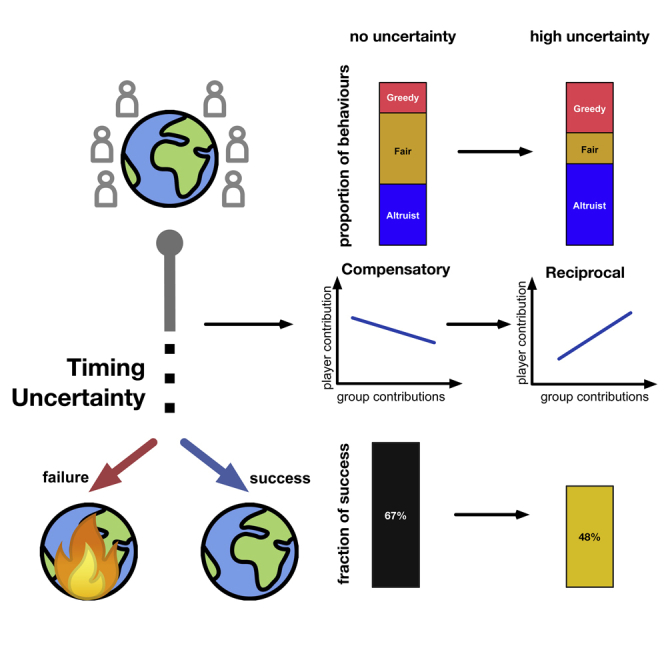

## Introduction

Public good games (PGGs) provide abstractions of many real-world problems wherein personal and short-term interests of multiple players are in conflict with societal, long-term interests (Perc et al, 2013, 2017; Ramamoorthy, 2013). Participants in such games can contribute voluntarily to a common good, which can, once established, be accessed without restrictions by all, thus even those that did not contribute. Rational selfish behavior stipulates that it is best to not contribute; yet such decision would be detrimental, as a group is better off when all contribute. These games not only serve as a good model for social public benefits (e.g., social security, retirement funds) but are also recurrent in many other collective endeavors, from group hunting (Hill, 2002; Kaplan et al., 2000) to public health (Brewer et al., 2007; Ferguson, 2007; Perisic and Bauch, 2009; Van Segbroeck et al., 2010; Westhoff et al., 2012) and socio-political processes like climate change (Abou Chakra and Traulsen, 2012; Barfuss et al., 2020; Barrett, 2016; Barrett and Dannenberg, 2012; Santos et al., 2012; Vasconcelos et al., 2013, 2015).

The current paper focuses on a variant of the PGG, i.e., the collective risk dilemma (CRD) (Milinski et al., 2008), where participants have multiple rounds to collect a target contribution, making the game non-linear, and the collective benefit uncertain as it is only achievable in the future. This model, part of a larger set of dilemmas also known as threshold PGG (Cadsby and Maynes, 1999; Offerman et al., 1998; Pacheco et al., 2009; Palfrey and Rosenthal, 1984), has been adopted to address the complexity pertaining decision-making under climate dilemmas (Abou Chakra and Traulsen, 2012; Chakra et al., 2018; Milinski et al., 2008; Pacheco et al., 2014), but its significance is general enough to be of interest to a broad range of human endeavors, such as costly signaling (Abou Chakra and Traulsen, 2012; Cimpeanu and Han, 2020; Gintis et al., 2001), voting (Kroll et al., 2007; Putterman et al., 2011), or petitioning (McBride, 2006; Pacheco et al., 2009). At the start of the game, participants are each given an endowment (*E*), and they must decide whether to contribute, up to a predefined amount, to the common good over a fixed number of rounds. If the joint contributions of all the participants over those rounds are equal or above a certain threshold, then the disaster is averted, and they receive as a reward the remainder of the endowment (hence the dilemma). On the contrary, if the target is not reached, there is a probability that a disaster may occur, resulting in economical loss for all the participants (they lose the remainder of their endowment). This is modeled by a risk parameter and both experiments and theoretical analysis show that people only tend to contribute to avoid the disaster if they perceive the risk to be high (Abou Chakra and Traulsen, 2012; Chen et al., 2014; Hagel et al., 2016; Milinski et al., 2008; Pacheco et al., 2014; Santos et al., 2012; Santos and Pacheco, 2011; Vasconcelos et al., 2015). Moreover, even when the risk is high, theoretical models indicate that players should delay their contributions when the moment of disaster is known (Abou Chakra and Traulsen, 2012; Hilbe et al., 2013).

Both threshold PGG and CRD make strong assumptions about what is known in the game: Each participant knows from the start how much they need to acquire collectively to reach the target and how much time they have to achieve this. Yet in real-world scenarios the amount as well as the timing when it has to be achieved may not be certain, as they are based on predictions and thus inherently suffer from uncertainties. Prior work on uncertainty about what amount (threshold) should be achieved in PGG (De Kwaadsteniet et al., 2007; Dijk et al., 2004) and CRD (Barrett and Dannenberg, 2012; Dannenberg et al., 2014) has shown that the level of cooperation, i.e., the willingness to contribute in both games, is negatively affected, yet no insights exist on how timing uncertainty affects the decision-making process.

To answer this question, three experimental treatments are performed here. First, as a control treatment (NU, no uncertainty), we investigated the behavior of groups of six participants, wherein each can contribute 0, 2, or 4 EMUs (Experimental Monetary Units) at each of the ten rounds of the experiment (*m*_0_ = 10). When the group does not reach the target contribution of 120 EMUs by the 10^th^ round, they risk losing the remainder of their initial endowment (40 EMUs) with a 90% probability. NU, thus, repeats the work of Milinski et al. (2008), but *without* the climate change framing, which avoids possible cultural effects due to climate awareness, while enabling the generalization of our conclusions to other problems captured by the CRD. Conversely, in the second treatment (LU, low uncertainty), participants did not know exactly when the experiment would finish. They were told the experiment lasts on average 10 rounds and that from round 8 there was a possibility that the game ends (see Figures S9 and S10 and the Transparent Methods in the Supplemental Information for instructions): a six-faces virtual dice was thrown, and the game would continue if the result was higher than 2 (i.e., the game ends with a probability of $w=1/3=1/\left[\left(10-m_{0}\right)+1\right]$). If the game continues, the same process is repeated at the end of each round, until the dice result indicated the end of the experiment. The game can thus stop early at round 8 but also continue multiple rounds after round 10. Finally, for the third treatment (HU, high uncertainty) we increased the uncertainty, i.e., the variance of the distribution of rounds, by making *m*_0_ = 6 (and *w* = 1/5—we throw a ten-faces virtual dice in this case). Importantly, we made sure that all participants are clearly informed, in every treatment, that the average number of rounds is 10 (their understanding was tested before starting the experiment). Note that in all three treatments, both with and without uncertainty, the threshold is always 120 EMUs and fair behavior corresponds to a contribution of, in total, half of one's endowment (*F* = *E*/2 = 20), as it would ensure that everyone has the same gains and that the target is met. When this target should be reached fully depends on the participants' beliefs.

## Results

### Impact of Timing Uncertainty on Group Success and Contributions per Round

**Figure 1:**
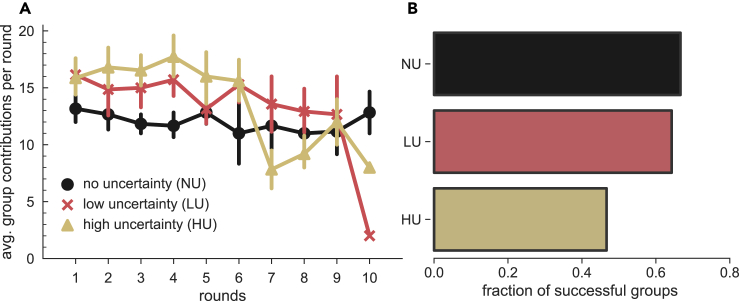
Average Group Contributions per Round and Fraction of Successful Groups (A) Average contributions per round and per group of 6 players for each treatment, with 95% CI bars. For NU (black lines, averaged over 12 groups), the groups contribute per round on average the minimum amount required to reach the target by the end of the game (12 EMUs per round). However, the contributions diminish slightly after round 5 and only recover at round 10. Treatments LU (red lines, averaged over 14 groups) and HU (yellow lines, averaged over 15 groups) show higher contributions early in the game, even though the game still takes on average 10 rounds: the higher the uncertainty on the timing, the higher the average amount contributed early in the game (see also Figures S1 and S2). However, these early contributions do not lead, on average, to a higher collective success. Instead, as shown in (B), one can see that the fraction of successful groups in HU is lower than those in NU and LU.

In the absence of any timing uncertainty (NU, black lines and bars in Figure 1), the experimental results show that groups can reach the target amount and that their contributions per round follow closely the minimum required to reach the target by the end of the 10^th^ round, i.e., an average of 12 EMUs per round (see Figure 1A). Figure 1B shows that more than 65% of the groups were successful in the NU treatment (fraction of successful groups, *η*). This success rate decreases with timing uncertainty especially in the HU treatment. Despite the lower success rate, whenever timing uncertainty is present (LU and HU), individuals tend to contribute earlier in the game, with the amount contributed in the first five rounds increasing with the uncertainty (see Figures 1A and S1 for the cumulated contributions). We have confirmed that this is the case for both groups that fail and succeed to achieve the target (see Figure S2 in the Supplemental Information).

This result appears to indicate that participants behave, on average, in a risk-averse fashion, trying to respond to the uncertainty by giving earlier to the collective dilemma. Yet, as one would expect, this only happens to the extent that the uncertainty does not require them to make excessive contributions from their own resources. For LU, participants contribute earlier, achieving success rates comparable with the case of NU. Differently, under HU, despite participants' reaction of further increasing their early investments, they are more hesitant to make even larger contributions in the first rounds, especially when they cannot be sure that others would do the same. As a result, lower success rates are achieved in the case of HU.

### Timing Uncertainty Increases Early and Polarized Contributions

Independent of when they try to reach the target, if every participant contributes half of his or her *E*, then the target would be reached and they all would go home with the same gains. This *fair share F* can be achieved by accumulating different combinations of 0, 2, or 4 EMUs over the different rounds. Indeed, we observe in the contributions per round for the NU experiment that most participants giving *F* do not do this by giving 2 in every round. Only 15% (11 of 72) of all participants giving *F* and 20% (10 of 48) of the successful ones do this by always giving what could be the locally fair share. Fairness in this game is thus defined at the game level and not the round level.

**Figure 2:**
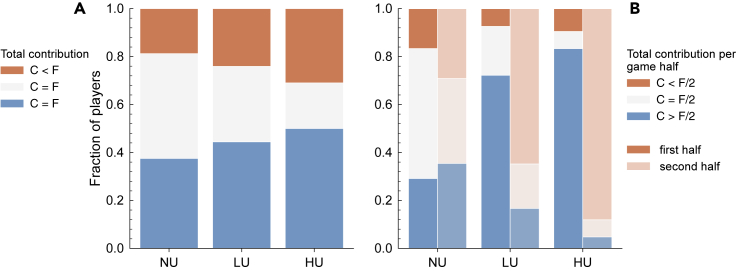
Distribution of Participant Behavior and Dependence on Time Fraction of players within successful groups that contribute, in total during the experiment, less than, equal to, or more than the fair contribution *F* (if all participants of a group contribute exactly *F*, they reach the target) (A) and how these behaviors are distributed over the first and second halves (1 half means 5, 4, and 3 rounds for NU, LU, and HU, respectively) of the game (B). (A) shows that as uncertainty increases, more players contribute either more or less than *F*, whereas the players that contribute exactly *F* decrease. (B) shows how this behavior is spread across the game, by showing the fraction of players that contribute less than, equal to, or more than *F*/2 in each half of the game. The plot shows that the contributions in the first half of the game increase with uncertainty, in comparison with NU (see also Figure S3).

Now, when considering the total contributions per participant during the experiment (*C*), one can observe that individuals react to uncertainty by either giving more or less than *F* (see Figure 2A). This suggests that the presence of timing uncertainty not only leads to earlier contributions, as mentioned earlier, but also generates polarized outcomes, i.e., unequal total contributions among participants, with more players' C deviating from *F* than when there is no uncertainty. This observation is confirmed in HU (see Figure 2A), where the prevalence of unfair contributions increases further when compared with NU and LU. These polarized outcomes may suggest again a co-existence of risk-averse and risk-seeking individuals (Higgins, 1997; Kahneman and Tversky, 1984), depending on whether individuals base their choices on a number of rounds below or above the average of 10 rounds.

The same cumulative data also reveal the shift toward early contributions when considering players' donations in the beginning and end of the game (see Figure 2B). The results show how participants appear to divide the game into two halves (from [1, *m*_0_/2] and [*m*_0_/2, *end*], i.e., half means 5, 4, and 3 for NU, LU, and HU respectively). The fraction of players whose C is more than, equal to, or less than half of the *fair contribution*, i.e., *F*/2, is shown for the groups that reached the target. In the treatments with uncertainty (LU and HU), the fraction of *C* > *F*/2 players in the first half is significantly higher than that of the second half, which means that participants contribute earlier to reach the target. In contrast, in NU there is a slight increase in *C* > *F*/2 during the second half of the game. This may be related to players trying to compensate for *procrastination,* resulting in higher contributions at the end. Moreover, when comparing the contributions between players that met and did not meet the target (see Figure S3 in Supplemental Information), the difference between the fraction of players that contribute *C* > *F*/2 in the first half of the game grows with uncertainty. This highlights the importance of contributing earlier and not *procrastinating* under the presence of timing uncertainty.

### Reciprocal Behaviors Emerge in Successful Groups under Timing Uncertainty

**Figure 3:**
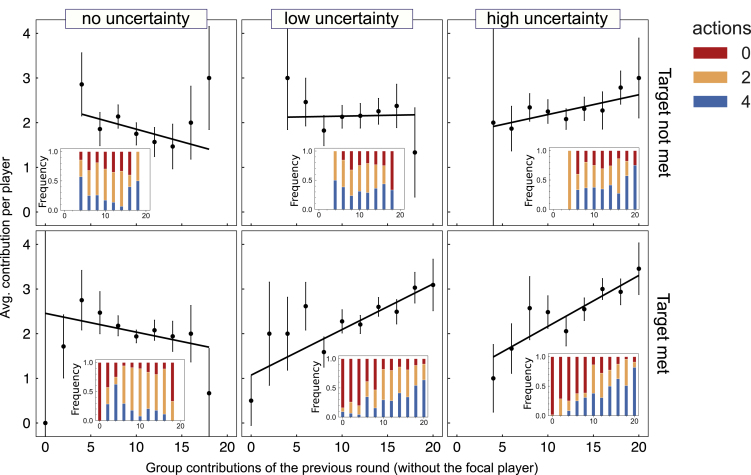
Prevalence of Group Reciprocity for Different Uncertainty Levels We show the average contribution per player (see Transparent Methods for details about the error bars and weighted linear regression) as a function of the contributions of the group members in the previous round (without the focal player). The plots are separated by treatment (columns) and by whether they met (True) or not (False) the target (rows). We fitted a weighted linear regression on each plot (see Figure S5 and Tables S5–S10 in Transparent Methods). This analysis shows that there is no significant dependency on the group contributions for NU, despite a slightly negative correlation factor for the players that reached the target, which indicates a compensatory behavior. However, there is a significant dependency on the group contributions under uncertainty (LU and HU). Moreover, for LU, the groups that did not reach the target display a slight compensatory behavior, in contrast to the reciprocal behavior of those that did. Inside each plot, we show a subplot of the frequency of each action (0, 2, and 4) for the different group contributions. These plots depict clearly how, for LU and HU, the frequency of action 4 increases with the group contribution on the previous round, whereas action 0 increases when the previous contributions were low. In comparison, action 2 is predominant when there is no uncertainty or when groups did not achieve the goal (LU-HU) (see also Figure S4 and Tables S1–S4).

Not only do participants tend to contribute earlier in the presence of timing uncertainty but also their contributions become dependent on what the group members did (see Figure 3), to the point that the predominant behavior in successful LU and HU resembles a group-level reciprocal behavior (Trivers, 1971) or Tit-for-Tat (TfT) (Axelrod and Hamilton, 1981; Van Segbroeck et al., 2012). Such group reciprocal behavior, or *group conditional cooperation*, has also been observed in linear public good games without a threshold (Chaudhuri, 2011; Fischbacher et al., 2001), and it has been identified experimentally as a beneficial strategy for climate action (Tingley and Tomz, 2014).

Although this behavior does not avoid free riding (there is actually an increase as can be seen in Figure 2A), it promotes generosity among the participants and appears necessary for a successful outcome. In Figure 3, we can see that there is a positive correlation between the group contributions and the average contribution of the players in LU and HU (see correlations in Tables S1–S4 in Supplemental Information) for the players that met the target. In contrast, the players that did not meet the target do not display the same conditional behavior or to the same extent, indicating that reciprocity is used here by the participants to achieve “honest” coordination in the presence of timing uncertainty (see Tables S5–S10 and Figure S5 in the Supplemental Information for an extended regression analysis).

The non-trivial dynamics and behavioral ecology of switching from compensating to reciprocal behavior may be explained by a game theoretical model that describes the behavioral dynamics through an evolutionary process (see Transparent Methods for a full description). Such model allows us to understand the shift in the distribution of behaviors in a population with and without timing uncertainty, while limiting the complexity of such an analysis to a minimum. As suggested by Figure 3, we mostly observe either unconditional contributions or conditional strategies based on the level of contributions in the previous round. We confirmed that individuals' tendency to contribute based on decisions taken previously by other group members (Van Segbroeck et al., 2012) also allows one to cluster the participants into different behavioral groups or strategic profiles (see Figure S4). These strategic patterns are associated with different degrees of compensatory and reciprocal behaviors, including also more classical give-all or give-nothing behaviors that provide contributions independent of the actions of the other participants in the group.

### Social Learning Model Confirms and Explains the Observed Behavioral Dynamics

**Figure 4:**
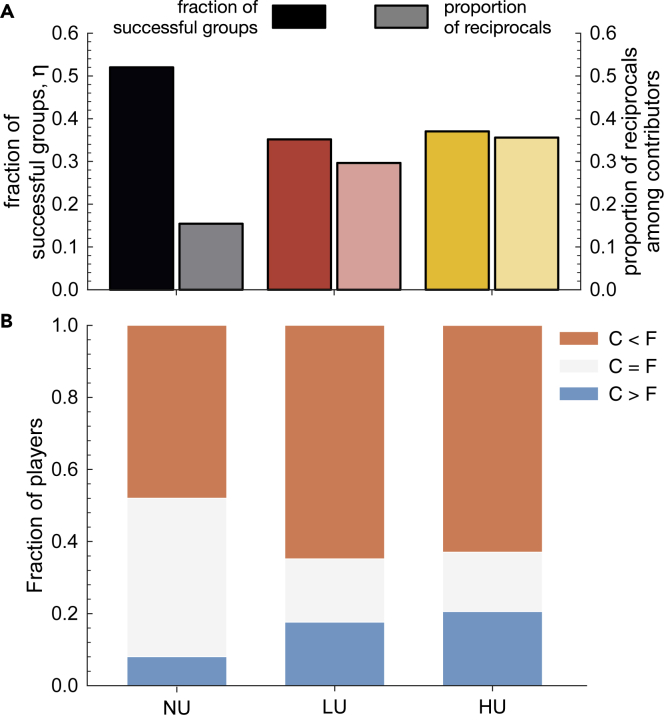
Emergence of Reciprocal Strategy and Polarization in a Stochastic Evolutionary Model (A) shows how the fraction of successful groups does not vary significantly with timing uncertainty (left y axis), while the predominance of the reciprocal strategy over the strategies that contribute to reach the target increases (right y axis and boxes with slashes). (B) shows that the polarization increases when there is timing uncertainty. These results reproduce the trend observed in the experimental data. (*Z* = 50, *N* = 6, *r* = 0.9, *E* = 40, *τ* = 120, *β* = 0.004, with *Z* being the population size, *N* the number of group members, *r* the risk of losing the remainder of the endowment when not reaching the threshold, *E* the initial endowment, *t* the threshold that needs to be achieved, and β the selection strength in the stochastic evolutionary dynamic. See Transparent Methods for a detailed explanation of this dynamic model and Figures S6–S8 for additional results).

Using this insight into the individual behaviors, we define a minimal game-theoretical model with three unconditional heuristics or strategies, i.e., *always-2* (gives 2 in every round), *always-4* (gives 4 in each round), *always-0* (contribute nothing throughout the game), and the two conditional ones, i.e., *compensator* (will contribute 4 when the group members did not contribute) and *reciprocal* (will contribute 4 as long as the group members contribute). All five strategies stop contributing once the collective target is achieved. We consider a population of individuals that may adopt one of these five strategies and revise their choices based on the relative success of each strategy (Traulsen et al., 2006) (see Transparent Methods for details). Despite its simplicity, this baseline model is shown to be sufficient to explain why both reciprocal behaviors and polarization increase with timing uncertainty. Indeed, the model confirms that, under uncertainty, the reciprocal strategy prevails among those strategies that contribute to the collective good (see Figure 4A), while capturing also that the fraction of successful groups does not change significantly with uncertainty. Moreover, the model indicates that the *always-2* strategy is only stable when there is no timing uncertainty (see Figures S6–S8). As detailed in the Supplemental Information, uncertainty transforms the game dynamics where free-riders and unconditional (fair) strategies dominate into a cyclic dynamic (akin to the famous rock-paper-scissor game), where the prevalence of reciprocal strategies increases with uncertainty. The model is also able to capture the inequality in contributions and the increase in polarization observed in the experiments (see Figure 4B).

## Discussion

Despite the simplicity of our experimental setup and associated theoretical model, the trends identified may hold potential lessons for dealing with uncertainty in local and global governance. Our results show that, contrary to the outcome for other types of uncertainty, timing uncertainty promotes early contributions by the participants as long as it does not conflict with the individual benefits that can be gained in the experiment. Moreover, timing uncertainty appears to increase polarized outcomes among the participants, suggesting a co-existence of risk-prone and risk-averse preferences, while diminishing the number of players contributing a fair share over their total resource and increasing those that give less or more. Interestingly, our result relates nicely with recent findings in the context of behavioral dynamics in urban settings. It has been shown that uncertainty associated with big cities intensified both risk-seeking and risk-taking reactions, whereas the predictability of small villages encouraged more homogeneous and intermediate choices (Ross and Portugali, 2018). Such heterogeneity highlights the intricacy of the study of the emergence of polarized behaviors beyond contagious processes (Centola, 2018), with potential implications in various socio-political and ecological contexts.

At the same time, conditional behaviors emerge in the presence of timing uncertainty, and groups that were able to coordinate through reciprocal behavior were more successful than those that simply played a fixed strategy or compensated for those giving not enough. This contrasts with the predominant behavior when the length of the game is certain, in which most players unconditionally opt to contribute a fair share *F*. Our results suggest that, when the future is uncertain and stakeholders are aware of it, they tend to respond early, whereas acting reciprocally appears to ensure groups to be successful. This effect may be potentially reinforced when combined with communication, institutions, or costly commitments (Han et al., 2017; Smead et al., 2014; Tavoni et al., 2011; Vasconcelos et al., 2013, 2015). The implications of these observations within the framing of real-world CRDs in health (Brewer et al., 2007; Ferguson, 2007; Perisic and Bauch, 2009; Van Segbroeck et al., 2010; Westhoff et al., 2012) and socio-political processes (Abou Chakra and Traulsen, 2012; Barfuss et al., 2020; Barrett, 2016; Barrett and Dannenberg, 2012; Santos et al., 2012; Vasconcelos et al., 2013, 2015), as mentioned above, may vary depending on the specific problem. We may nonetheless highlight that, in light of our results, uncertainty on, e.g., the urgency of reducing CO_2_ emissions or the risk of a pandemic, may trigger reciprocal behaviors and further reinforce polarization, with potential detrimental impacts that can be hardly overemphasized.

### Limitations of the Study

We have performed controlled behavioral economic experiments in a laboratory with human participants. Although our study adheres to all standards of behavioral economics research, the subject pool is limited to university students located in Brussels. Demographics is thus limited to a specific age group. Future work should consider expanding our experimental pool. The game-theoretical model we employ relies on population dynamics and social learning (Sigmund, 2010) and is capable of capturing the conditions in which timing uncertainty induces a shift toward reciprocal behaviors and polarization. However, this is one among many approaches that could be used (see, e.g., Barfuss et al., 2020; 2017; Bloembergen et al., 2015, for alternatives). Having multiple models based on different assumptions confirming the same observations can only strengthen the results and improve our understanding of these complex self-organizing phenomena. Finally, models with more elaborate representations of strategies and individual behaviors can further enrich our understanding on the emergence of specific collective patterns and behavioral profiles. Representations capturing, for instance, the choices of participants at each step of the CRD would allow for more fine-grained categorizations and more specific comparisons with the participants' behaviors in the experiments. Here, we adopted a model with a level of complexity that can be justified by the dataset obtained from laboratory experiments; further expansions of this model may require the support from more detailed empirical observations, and, for this reason, are left for future work.

### Resource Availability

#### Lead Contact

Further information and requests for resources should be directed to and will be fulfilled by the Lead Contact, Tom Lenaerts (tlenaert@ulb.ac.be).

#### Materials Availability

No materials were newly generated for this paper.

#### Data and Code Availability

• The dataset generated and analyzed during the current study has been deposited at Dryad Data and can be cited as: Fernández Domingos, 2020b Data from: Timing uncertainty in collective risk dilemmas encourages group reciprocation and polarization, Dryad, Dataset, https://doi.org/10.5061/dryad.5qfttdz2t.
• The code for evolutionary dynamics in finite populations, used to produce the theoretical results is available at https://doi.org/10.5281/zenodo.3687125. We use the EGT framework from https://github.com/Socrats/EGTTools (Fernández Domingos, 2020a). Moreover, the manuscript Methods text, the figure captions, and the Supplemental Information provide all the parametric details for recreating the theoretical results.

## Methods

All methods can be found in the accompanying Transparent Methods supplemental file.

## Supplemental Information

Document S1. Transparent Methods, Figures S1–S10, and Tables S1–S10

## Footnotes

Supplemental Information can be found online at https://doi.org/10.1016/j.isci.2020.101752.
