# Supplementary material for: Timing Uncertainty in Collective Risk Dilemmas Encourages Group Reciprocation and Polarization
Source: iScience. 2020 Oct 31;23(12):101752. doi: 10.1016/j.isci.2020.101752 (PMC7701182; doi:10.1016/j.isci.2020.101752)
Supplement: Document S1. Transparent Methods, Figures S1–S10, and Tables S1–S10 [file mmc1.pdf]

iScience, Volume *23*

Supplemental Information

Timing Uncertainty in Collective Risk Dilemmas Encourages Group Reciprocation and Polarization

Elias Fernández Domingos, Jelena Grujić, Juan C. Burguillo, Georg Kirchsteiger, Francisco C. Santos, and Tom Lenaerts

Supplemental Information

1. Supplemental figures
2. Supplemental Tables
3. Transparent methods
4. Supplemental references

1. Supplemental Figures

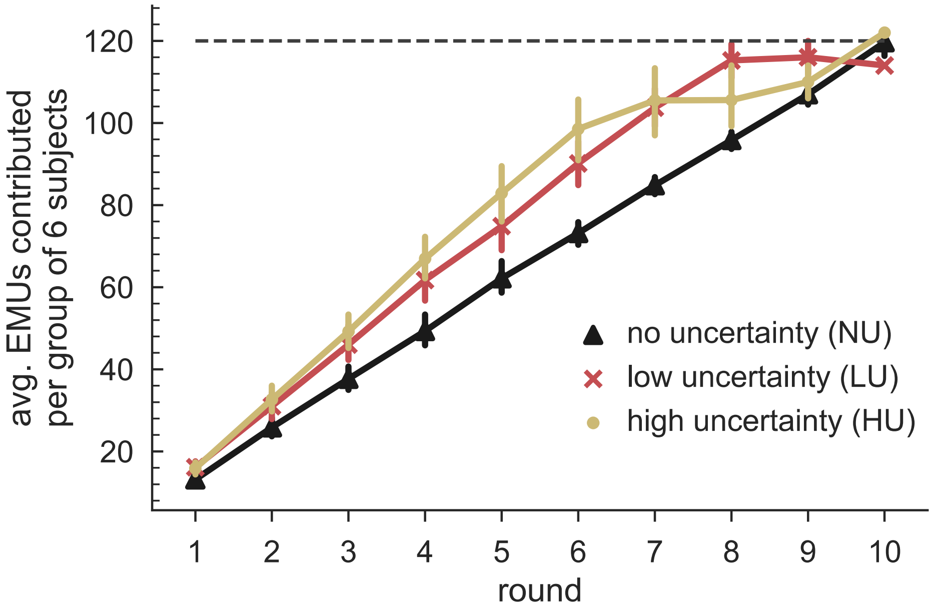

Figure S1. Average accumulated group contributions per round and treatment, related to Figure 1A. The x axis indicates the rounds of the game, while the y axis indicates the total average amount accumulated in the public account. The results are separated by treatment, and show a clear increase in earlier contributions for the treatments with uncertainty (LU and HU). By round 10, the target is achieved on average in all treatment. However, on the LU (low uncertainty) treatment, contributions already surpass 120 EMUs (the target) on average by round 8. At each point, the accumulated contribution is averaged only among groups which did not reach the target already on the previous round.

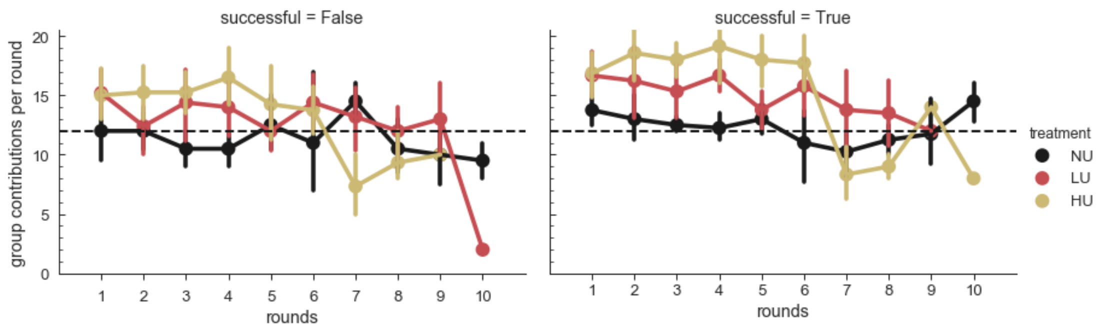

Figure S2. Average joint contribution per round and per group, related to Figure 1A. The plots are separated by whether the group reached or not the target collective investments. The dashed lines show *the* fair sum of contributions per round if the game had 10 rounds for each of the treatments (black – NU, red – LU, yellow – HU). We only show the contributions before the target is reached.

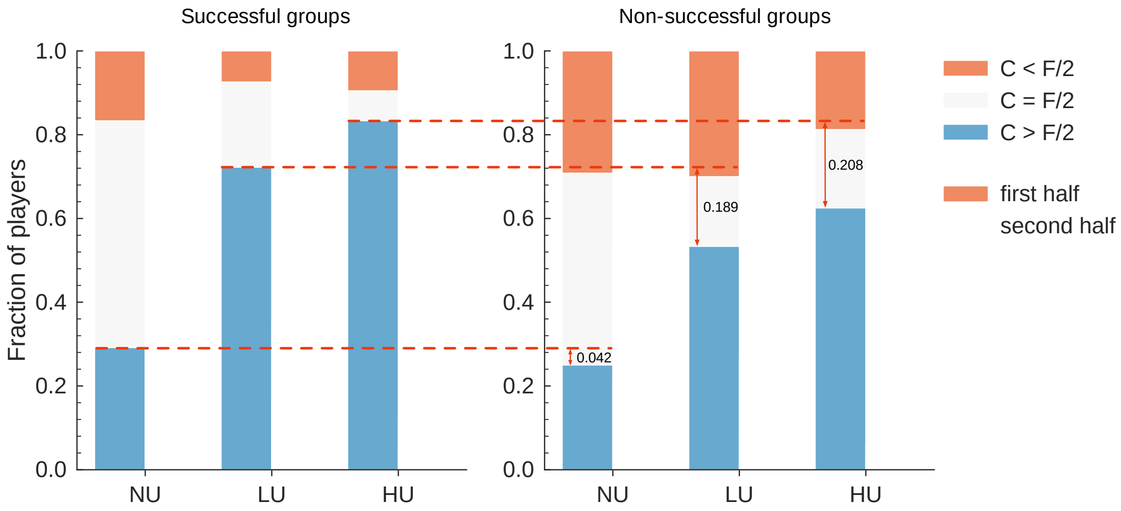

Figure S3. Distribution of players according to their contributions. The plots are separated by whether the groups reached (successful) or not (non-successful) the target, related to Figure 2B. The plots show the fraction of players that contributed more, equal or less than $F/2$, i.e., half of the *fair donation*, in the first and second half of the game. If every player contributed in total F. during the game, the group would reach the target with exactly 120 EMUs. For T2 and T3 we consider half of the game to be $m_0/2$. We can observe that the number of participants that contribute more than $F/2$ in the first half of the game (non-procrastinators), increase considerably in the treatments with uncertainty. It is also noticeable that the difference in the fraction of non-procrastinators between the groups that met and did not meet the target, increases with uncertainty.

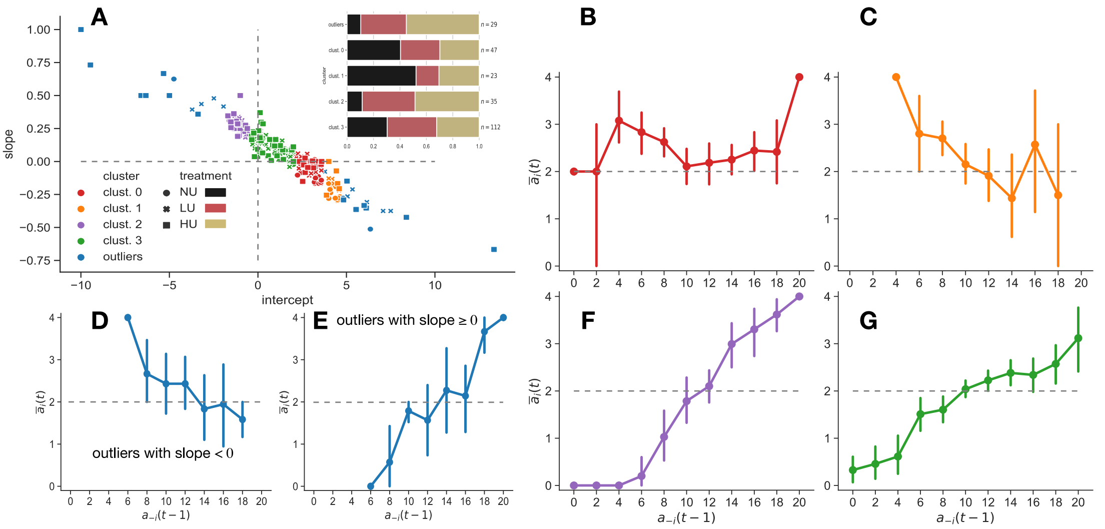

Figure S4. Behavioural clusters identified within the set of all participants, related to Figure 3. In panel (A), the behaviour of each participant in the experiment (including all three treatments) is represented by a slope and an intercept. These parameters are obtained by regressing linearly the average contribution of each participant at a given round, $\overline{a_i}(t)$, in function of the total contribution of the groupmates in the previous round, $a_{-i}(t)$. These points are then clustered using a DBSCAN algorithm, which identifies 4 clusters and a set of 29 outliers (optimised parameter $eps = 0.17$). The proportion of players of each treatment in the clusters is represented in the inset of this panel. Panels (B), (C), (F) and (G) show the averaged contributions of players in each cluster in response to $a_{-i}(t)$. Players in cluster 0 (panel (B)) display an almost unconditional response, and always contribute slightly above 2 EMUs. In contrast, players in cluster 1 (panel (C)) are slightly compensatory, making higher contributions when the group fails to contribute enough. The players represented in these two clusters are more predominant in NU. Contrarily, players in cluster 2 (panel (F) belong mostly to LU and HU. This cluster exhibits a strong reciprocal response, with the average contribution of players being directly proportional to $a_{-i}(t)$. Players in cluster 3 (panel (G)) belong in almost equal proportion to all 3 treatments, and they represent a strict fair behaviour, i.e., players contribute 2 EMUs unless the contributions of the group are too low. Finally, the set of 29 outliers is composed mostly of players of the LU and NU treatments and display opposing conditional behaviours (either compensatory – panel (D) – or reciprocal – panel (E)).

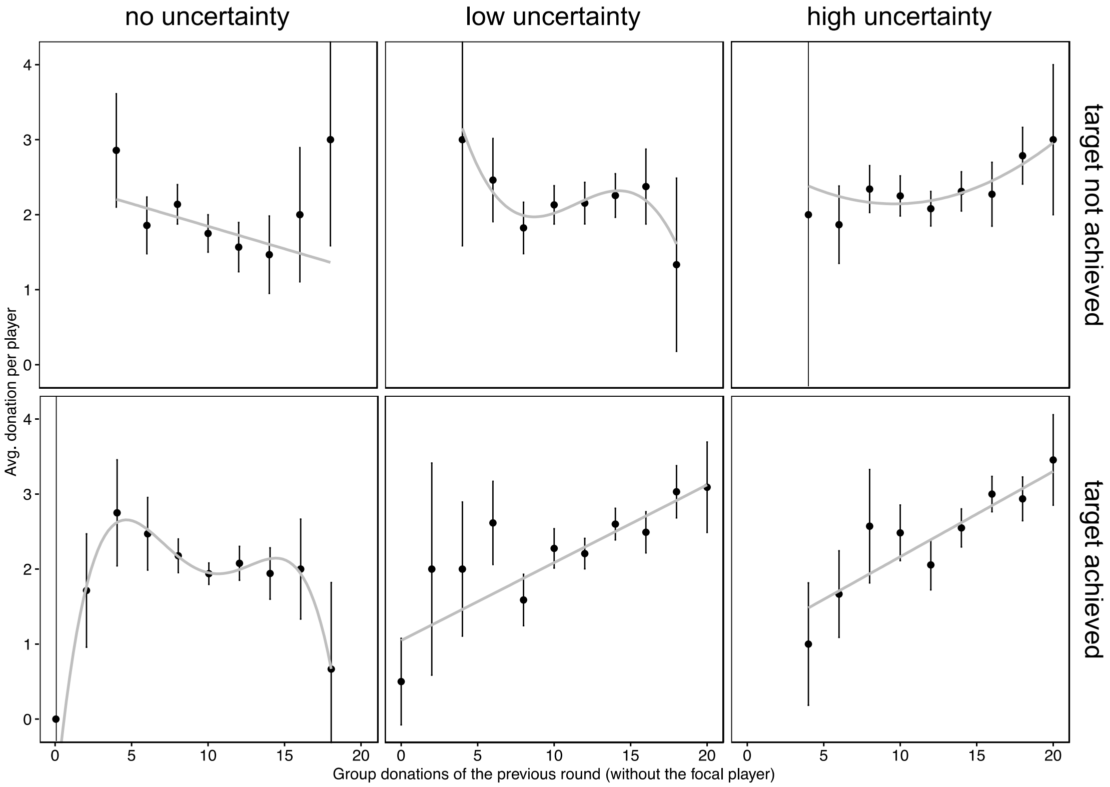

Figure S5. Analysis of conditional behaviour using the best fitting model for each case, related to Figure 3. Applying the best fitting model identified through our tests to each treatment does not change our conclusions, but highlights that on the certainty treatment, when players meet the collective target, they display a slight compensatory behaviour, while considerably lowering their contributions when the rest of the group adopt extreme actions: they donate either too much or too little.

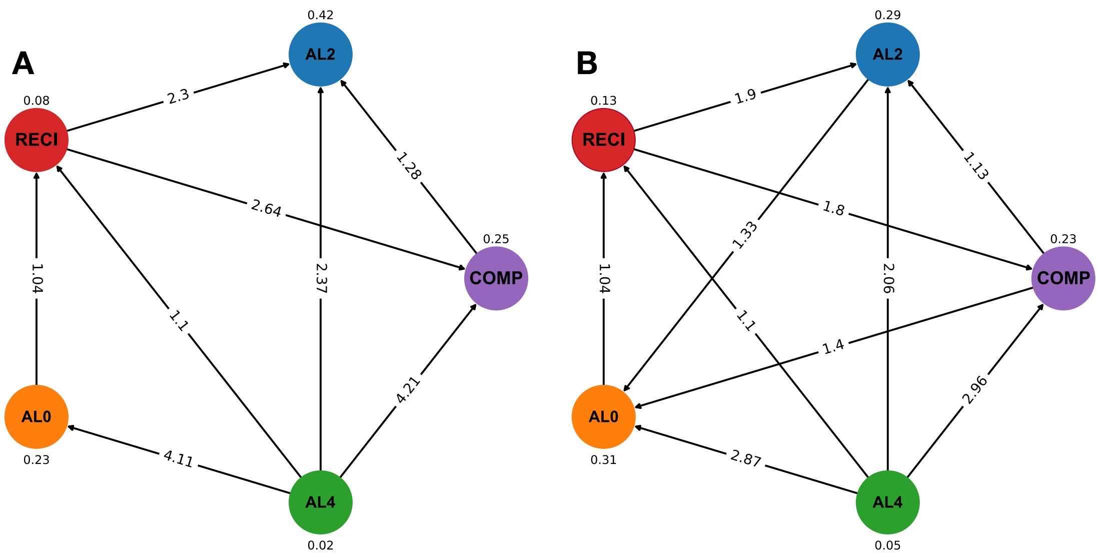

Figure S6. Markov chain depicting the transition probabilities between states, related to Figure 4. In panel (A) we show the Markov chain when there is no timing uncertainty. An arrow that goes from state i to j indicates that a population in state i (where all members of the population are of strategy i) will transition to strategy j with a probability higher than random drift. The number on top of each state indicates the stationary distribution, i.e., the time the population spends in that state. When there is no timing uncertainty, *always-2* is an evolutionary stable strategy (all arrows point to it, and none goes out). However, in the high timing uncertainty case (Panel (B)), it becomes dominated by *always-0.* Also, *reciprocal* strategy weakly dominates *always-0*, resulting in a cyclic dynamic (no strategy dominates). This explain the reduction in *always-2* players and the increase of *reciprocals* ($\beta = 0.004$, $Z = 50$).

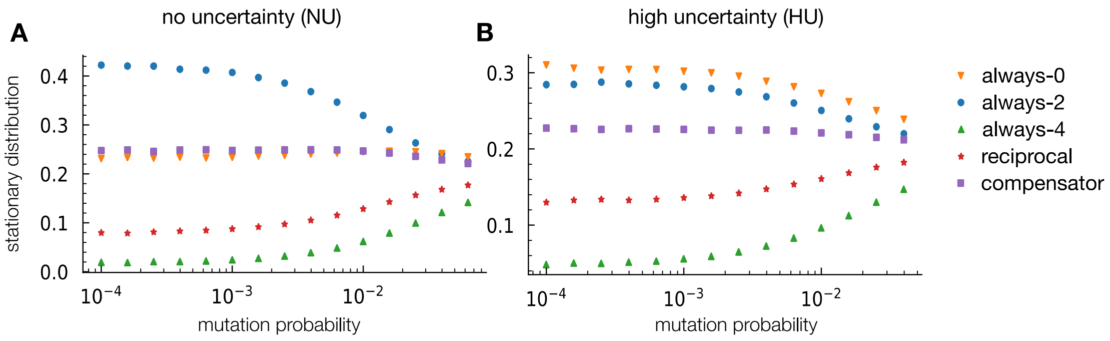

Figure S7. Influence of noise (mutation) in the stationary distribution, related to Figure 4. This figure shows how the stationary distribution of the monomorphic states (only one strategy in the population) is affected by the mutation probability (the probability that a random mutant appears in the population). The results show that the small mutation assumption in the theoretical model is valid for a large value of mutation probabilities both in the case of no uncertainty (Panel (A)) and high uncertainty (Panel (B)). Only for mutation values higher than 10^{-2} does the distribution get significantly affected. In this case, mixed states (more than one strategy survives in the population) become more common ($\beta = 0.004$, $Z = 50$).

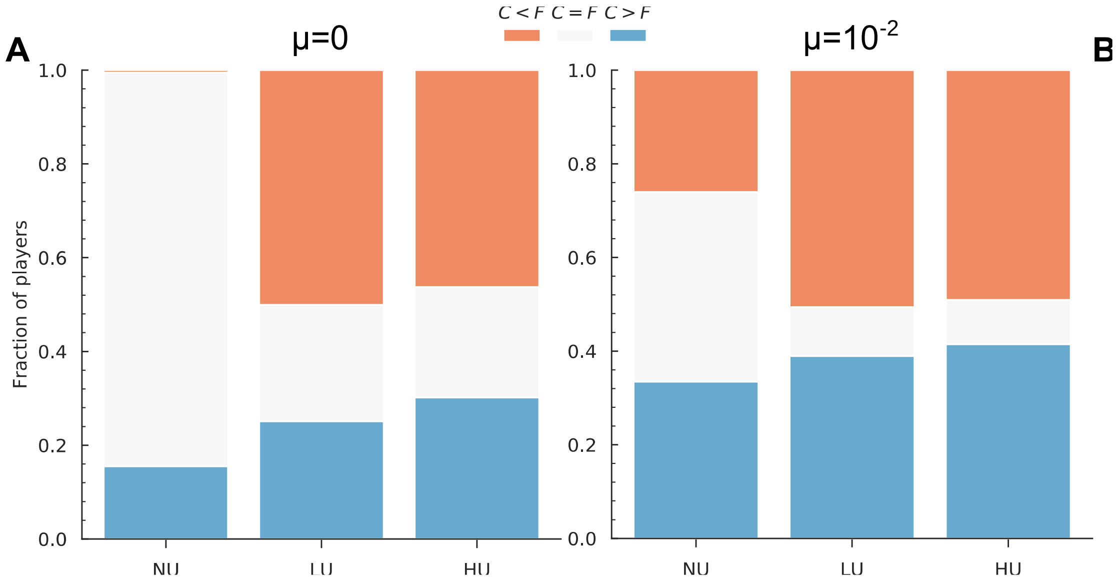

Figure S8. Distribution of contributions when only considering successful groups, related to Figure 4. In panel (A) we show the fraction of the population that contributes more, less or equal to the fair donation (*F*), considering only groups that achieve the target. We can observe that, while in the no uncertainty (NU) case, successful groups either contribute equal or above F, an important fraction of the population contributes below F in the timing uncertainty cases. This indicates the emergence of polarization with timing uncertainty, as we observed in the experiments. In panel (B), we show the same results, but considering a mutation rate $\mu = 10^{-2}$. Here, our model' results matches closely the experimental results for NU, while it predicts an even more extreme case of polarization for LU and HU ($\beta = 0.004$, $Z = 50$).

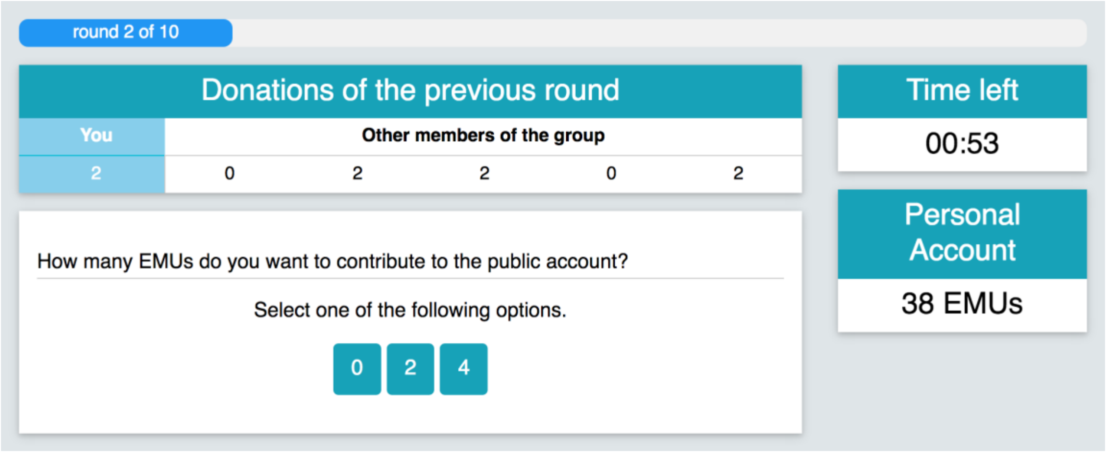

Figure S9. View of Step 1 of the experiment shown in the experimental instructions. In this step participants have to decide which contribution (0, 2 or 4 EMUs) to make.

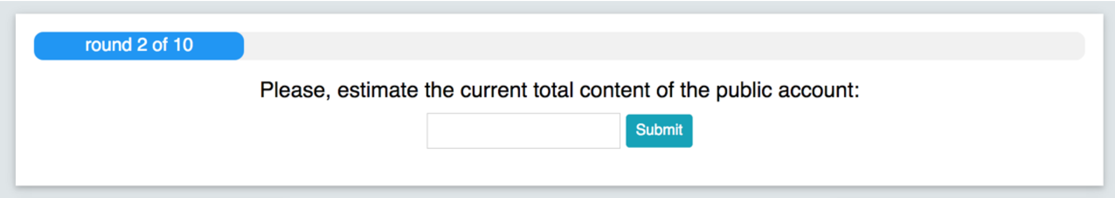

Figure S10. View of Step 2 of the experiment shown in the experimental instructions. In this step, participants have to estimate how much has been contributed to the public account in total, including the contributions of the last round. Thus, participants have to predict how much was contributed in the last round, since this information is not yet known in this step.

2. Supplemental Tables

Table S1. Correlation between behaviour of others and the behaviour of a focal player, related to Figure 3. This table shows the Pearson correlation coefficients and the associated p-value between the sum of donations of the other members of a group in a previous round and the action of the focal player on the current round. These results are associated with Figure 3 of the main text. The correlation is positive ($P < 0.001$) for the successful players (target = TRUE) on LU and HU, while the ones that failed to reach the target have a correlation close to 0. The players in NU have a small negative correlation that indicates the presence of *compensating* behaviours.

treatment	target	correlation	p-value	Number of observations
NU (No uncertainty)	FALSE	-0.117	0.086	216
	TRUE	-0.076	0.114	432
LU (Low uncertainty)	FALSE	0.019	0.775	228
	TRUE	0.279	< 0.001	408
HU (High uncertainty)	FALSE	0.092	0.114	300
	TRUE	0.268	< 0.001	282

Table S2. Ordered logistic regression for the NU treatment, related to Figure 3. This table shows how much each feature influences the selection of an action for a player. The p-value threshold selected here is $p<0.05$. The results highlight that, among others, time (GameHalf) is a relevant feature, however, in the donation of the other members of the group (round_donations_others) does not influence significantly the selection of an action.

Coefficients	Value	Std. Error	t value	p value
public_account	-0.0370	0.0181	-2.0436	0.0410
private_account	-0.2503	0.0969	-2.5822	0.0098
round_donations_others	0.0656	0.0650	1.0091	0.3129
actions_prev	0.3860	0.1749	2.2076	0.0273
GameHalf2ndHalf	-13.4311	4.2979	-3.1251	0.0018
rnd	0.0510	0.1660	0.3074	0.7585
public_account:GameHalf2ndHalf	0.0583	0.0204	2.8645	0.0042
round_donations_others:GameHalf2ndHalf	-0.2008	0.0765	-2.6266	0.0086
actions_prev:GameHalf2ndHalf	0.0642	0.2009	0.3194	0.7494
private_account:GameHalf2ndHalf	0.3827	0.1042	3.6719	0.0002

Intercepts				
0|2	-10.1362	3.9585	-2.5606	0.0104
2|4	-6.7677	3.9472	-1.7146	0.0864

Residual Deviance:	1084.3870
AIC:	1108.3870

Table S3. Ordered logistic regression for the LU treatment, related to Figure 3. The results for low uncertainty show that, like for no uncertainty, time is important. However, in this case, the donations of the other participants (round_donations_others) are also significantly affecting the action selection.

Coefficients	Value	Std. Error	t value	p value
public_account	-0.0556	0.0090	-6.1725	< 0.0001
private_account	-0.4054	0.0199	-20.3623	< 0.0001
round_donations_others	0.1178	0.0405	2.9103	0.0036
actions_prev	-0.1736	0.0955	-1.8177	0.0691
GameHalf2ndHalf	-11.4270	0.5859	-19.5022	< 0.0001
rnd	0.0986	0.1436	0.6867	0.4923
public_account:GameHalf2ndHalf	0.0130	0.0095	1.3606	0.1737
round_donations_others:GameHalf2ndHalf	-0.0140	0.0471	-0.2961	0.7672
actions_prev:GameHalf2ndHalf	0.3034	0.1132	2.6803	0.0074
private_account:GameHalf2ndHalf	0.3342	0.0199	16.7546	< 0.0001

Intercepts				
0|2	-16.4973	0.6035	-27.3366	< 0.0001
2|4	-14.3607	0.5973	-24.0445	< 0.0001

Residual Deviance:	1419.8400
AIC:	1443.8400

Table S4. Ordered linear regression for the HU treatment, related to Figure 3. Similar to the low uncertainty case, for high uncertainty we can observe that both time and the donations of other in the group are relevant features.

Coefficients	Value	Std. Error	t value	p value
public_account	-0.0728	0.0189	-3.8568	< 0.0001
private_account	-0.3897	0.0218	-17.8795	< 0.0001
round_donations_others	0.1503	0.0568	2.6442	0.0082
actions_prev	0.0886	0.1039	0.8532	0.3936
GameHalf2ndHalf	-12.0490	0.5227	-23.0520	< 0.0001
rnd	-0.1145	0.1614	-0.7098	0.4779
public_account:GameHalf2ndHalf	0.0347	0.0192	1.8070	0.0708
round_donations_others:GameHalf2ndHalf	0.0121	0.0621	0.1956	0.8449
actions_prev:GameHalf2ndHalf	0.2040	0.1210	1.6852	0.0920
private_account:GameHalf2ndHalf	0.3167	0.0224	14.1248	< 0.0001

Intercepts				
0|2	-15.4816	0.5294	-29.2444	< 0.0001
2|4	-13.6380	0.5254	-25.9587	< 0.0001

Residual Deviance:	1145.2530
AIC:	1169.2530

Table S5. ANOVA test for the NU treatment, and players that did not meet the target, related to Figure 3. No higher order polynomial model performs significantly better than the linear fit.

Model	Res.Df	RSS	Df	Sum of Sq.	F	Pr(>F)
1	6	3.6549				
2	5	2.3961	1	1.25873	3.1757	0.1728
3	4	1.867	1	0.52912	1.3349	0.3316
4	3	1.1891	1	0.67793	1.7104	0.2821

Table S6. ANOVA test for the NU treatment, and players that met the target, related to Figure 3. A fourth order polynomial generates a significantly better fit than the linear model.

Model	Res.Df	RSS	Df	Sum of Sq.	F	Pr(>F)	
1	8	3.3276					
2	7	3.2601	1	0.06752	0.6832	0.44611	
3	6	3.2579	1	0.00219	0.0222	0.887473	
4	5	0.4941	1	2.76378	27.9654	0.003223	**

Table S7. ANOVA test for the LU treatment, and players that did not meet the target, related to Figure 3. A third order polynomial provides a better fit than the linear model.

Model	Res.Df	RSS	Df	Sum of Sq.	F	Pr(>F)	
1	6	2.37502					
2	5	2.277	1	0.09802	0.377	0.58265	
3	4	0.78189	1	1.49511	5.75	0.09605	.
4	3	0.78006	1	0.00183	0.007	0.9385	

Table S8. ANOVA test for the LU treatment, and players that met the target, related to Figure 3. No model provides a better fit than the lineal model.

Model	Res.Df	RSS	Df	Sum of Sq.	F	Pr(>F)
1	9	6.7944				
2	8	6.6108	1	0.18354	0.2259	0.6514
3	7	5.6696	1	0.94127	1.1584	0.3232
4	6	4.8753	1	0.79422	0.9774	0.361

Table S9. ANOVA test for the HU treatment, and players that did not meet the target, related to Figure 3. A second order polynomial provides a better fit than the linear model.

Model	Res.Df	RSS	Df	Sum of Sq.	F	Pr(>F)
1	7	2.22619				
2	6	1.49467	1	0.73152	5.9811	0.07078 .
3	5	0.93891	1	0.55576	4.5441	0.10002
4	4	0.48922	1	0.44969	3.6768	0.12764

Table S10. ANOVA test for the HU treatment, and players that met the target, related to Figure 3. No model provides a better fit than the linear model.

Model	Res.Df	RSS	Df	Sum of Sq.	F	Pr(>F)
1	7	3.6				
2	6	3.5877	1	0.01231	0.0191	0.8969
3	5	3.2477	1	0.33999	0.5262	0.5084
4	4	2.5846	1	0.66316	1.0263	0.3683

3. Transparent Methods

Nomenclature

We refer to each one of the 3 treatments analysed in this manuscript in the following way:

- Treatment 1: no uncertainty treatment – NU
- Treatment 2: low uncertainty treatment – LU
- Treatment 3: high uncertainty treatment - HU

Experimental procedure

During each session of the experiment, all participants were required to read the instructions on the screen of their assigned computer before the start. The same instructions were provided on a printed copy that they could consult throughout the experiment. After reading, all participants went through a test with the goal to check their understanding. In case of problems the coordinators discussed with them their errors ensuring that everything was clear.

Throughout the experiment each participant observed on his or her screen the amount left in his or her *private account* and the actions of each of the group members in the previous round. They are thus able to keep track of the behaviour of their group mates, but do not know their identity. They could not observe the current state of the *public account*, yet, they were encouraged to keep track of it by asking them how much they believe is in the account at each round. After the final round (in the treatment with uncertainty they could observe the result of the random value produced by the dice that defined the end of the game), the participants could see on the screen how much was contributed in the *public account* in total and how much was left in their *private account*, as well as the conversion to euros. In the case this value was below the target, a message would show the result of the dice that decided whether they would lose or not the remaining endowment. Finally, before the participants were allowed to leave the laboratory, they were requested to complete a small survey about their experience during the experiment.

Our experiment models the effect of timing uncertainty on the collective-risk dilemma. Therefore, there is a stochastic component that we must explain to participants carefully. In order to do this, we used the known example of a virtual dice. For instance, to explain that there is a probability of 1/3 that the game would finish after round 8 in the low uncertainty (LU) treatment, we explain that the computer will “throw a virtual dice of 6 faces, and if the result is either 1 or 2, then the game will end”. We also tell participants that, on average, the game takes 10 rounds, to give them an intuition about the distribution of this stochastic process. The details of the instructions for all three treatments can be found in the next section (Experimental instructions).

Experimental instructions

<u>Instructions for the control treatment (no uncertainty – NU)</u>

Each participant had access to the following instructions, both in digital and paper format:

Instructions to the experiment

Welcome to this experiment where you can earn money!

You are about to participate in an experiment on iterative decision-making, conducted by researchers from the *Vrije Universiteit Brussel* and the *Université Libre de Bruxelles*. In this experiment, you will earn some money, and the amount will be determined by your choices and the choices of the other participants.

Your privacy is guaranteed: The other participants will not know who you are during the experiment and the results of the experiment are stored in an anonymous manner.

It is very important that you remain silent during the whole experiment, and that you never communicate with other participants, neither verbally, nor in any other way. When in doubt or when you have a question, please just raise your hand and an experimenter will approach you. If you do not remain silent, or if you behave in any way that could potentially disturb the experiment, you will be asked to leave the laboratory, and you will not be paid.

All your earnings during the experiment will be expressed in Experimental Monetary Units (EMUs), which will be transformed into Euros with a change rate of 0.75 Euro to 1 EMU. At the end of the experiment, a show up fee of 2.5 euros will be added to your earnings.

You will be paid privately by bank transfer to your account within a week after the experiment. At the end of the experiment you will be requested to provide your **IBAN number and BIC code** to make the transfer.

Before starting, you will be randomly assigned into a group. You will never know the identity of the other participants of the group. However, the experiment takes 10 rounds and you will be able to observe the actions of the previous round of every member of your group, starting from round 2.

Login to the experiment

Before the experiment can start, please, enter the user login and password you have been given into the login page displayed in the browser of the computer assigned to you.

Once you have logged in, you will be able to see on your screen the same instructions that are written on this paper.

Wait for the instructor's signal before you proceed.

General Information

At the beginning of the experiment you will be randomly assigned to a group, which will include 5 other randomly selected participants.

During the whole experiment, you will interact only with those 5 other group members.

At the beginning of the experiment you and each other group member will receive **a personal endowment of 40 EMUs**.

The whole experiment consists of 10 rounds of the following game.

In each round of the game, you have to decide whether to add 0, 2 or 4 EMUs in a **public account**.

If the public account contains at least **120 EMUs** after the 10th round, **each member of your group will keep their savings**, i.e. the EMUs of your endowment that were **not** put in the public account.

However, **if this minimum is not reached,** the computer will "throw a virtual dice" and **each group member will lose his or her remaining EMUs with a 90% chance (9 times out of 10)**.

Thus, with a 10% chance (1 out of 10) you will keep the remaining EMUs in your private account.

Course of Action

Every round has the same structure and consists of the following **steps**:

Step 1:	Choice of how much to contribute (0,2 or 4).
Step 2:	Make a prediction about the amount in the public account.

When the experiment reaches its final round, you will move to the final 3rd step:

Step 3: Check if the threshold of the public account has been achieved and calculation of final payoffs

Step 1: The contribution choice

In the Step 1, every member will be asked **"How many EMUs do you want to contribute to the public account"**. Three buttons are provided: **0, 2 and 4 EMUs**. You can select the amount by clicking the button, as is shown in the figure below: (see Figure S9)

On the right side of the screen you can see the time you have left to make your decision and the amount of EMUs in your **"Personal Account"**. **You must make your decision within the time displayed on the screen**. The "Time left" square will start blinking when you are getting out of time. Nothing happens when the time runs out, yet if you take too long to make a decision the experiment will take too long. Please respond as quickly as possible.

The table "Donations of the previous round" shows the values donated by all the members of **your group** in the previous round. In the first column, you see **your own donation** from the previous round. In the other columns, you see the decisions of the other users. The choice of each group member will always be shown in the same column. This information about the previous donations is only available after the first round.

Step 2: Predict the content of the public account

After step 1, you will go to a next screen. On this screen, you are asked the following question: "**Please, estimate the current total content of the public account**". You should enter an estimation of how many EMUs you think the **public account** contains **in total** after all members (including you) have made their donations in the current round.

This is an example of what you will see in this step: (See Figure S10)

Step 3: Last round and calculation of final payoffs

After the last round, you will jump to a final screen.

If the accumulated contributions to the account are equal or higher than **120** EMUs, then you will be informed that you can keep the amount of the endowment that you **did not** put in the public account.

For example, if you put in total 20 EMUs of your endowment (40 EMUs) in the public account during the experiment, you will gain the remaining **20** EMUs (i.e. 40 - 20). This amount is converted into Euro's.

The screen will show the following text: "*CONGRATULATIONS! Your group collected XXX EMUs, which is greater or equal to 120 EMUs. So* ***you may keep the amount remaining in your private account****. Please fill in the amount in Euro's you see on this screen on the payment document you received before clicking the continue button. This amount consists of both your private winnings and the show-up fee."*

However, **if the minimum of 120 EMUs is not reached,** the computer will "throw a virtual dice" and **all group members will lose all their remaining EMUs with a 90% chance.** There are thus two possible outcomes:

On one hand, **with a chance of 9 out of 10, the screen will show**: "*Your group collected XXX EMUs, which is lower than 120 EMUs. The server has generated a random number between 1 and 100. The resulting value is YYY, which is smaller than 91. This means that you all* ***lose the remaining endowment*** *in your private accounts. Please fill in the amount in Euro's you see on this screen on the payment document you received before clicking the continue button. This amount is the show-up fee.*"

On the other hand, **with a chance of 1 out of 10, the screen will show**: "*Your group collected XXX EMUs, which is lower than 120 EMUs. The server has generated a random number between 1 and 100. The resulting value is YYY, which is bigger than 90. This means that you all* ***win the remaining endowment*** *in your private accounts. Please fill in the amount in Euro's you see on this screen on the payment document you received before clicking the continue button. This amount consists of both your private winnings and the show-up fee.*"

<u>End of experiment questionnaire</u>

At the end of the experiment you will be directed to a form containing a short questionnaire. Please answer to all the questions honestly, the information you add here is an important part of this experiment. Any information that you may include in this form will remain completely anonymous and cannot be linked to you in any way. Once you have finished filling in the questionnaire, please, click the button ***submit***.

At the end of the experiment, you will be called by one of the organisers to make the payment official. Please stay seated and do not talk until you are called and have left the room.

<u>Please note:</u>

Communication is not allowed during the whole experiment. If you have a question, please raise your hand.

All decisions are made anonymously, i.e. no other participant learns the identity of the other decision makers.

The payment is also anonymous, no participant learns from us about the amount that another participant received in the experiment.

<u>Instructions for the low uncertainty treatment (LU)</u>

The instructions of the treatment with low *timing uncertainty* differ slightly from that of the previous treatment. Below we describe only the sections that change with respect to the control treatment (NU).

Instructions to the experiment

Before starting, you will be randomly assigned into a group. You will never know the identity of the other participants of the group. However, the experiment takes **at least** 8 rounds and you will be able to observe the actions of the previous round of every member of your group, starting from round 2.

<u>General Information</u>

The whole experiment consists **of minimum 8 and on average 10 rounds, but it could be more than that.**

The probability that the next round will happen after round 8 is 2/3. This means that at the end of each round, starting from round 8, a **"virtual fair dice"** with 6 faces, will be thrown. If the result is **"1"** or **"2"**, the game will end. Otherwise, when the result is "**3**", "**4**", "**5**" or "**6**", the game will continue to the next round, in which the process is repeated.

If the public account contains at least **120 EMUs** after the final round, **each member of your group will keep their savings**, i.e. the EMUs of your endowment that were **not** put in the public account.

<u>Course of Action</u>

Every round has the same structure and consists of the following **steps**:

Step 1: Choice of how much to contribute (0,2 or 4).
Step 2: Make a prediction about the amount in the public account.

The end of the experiment is decided by a random process. Starting from **round 8** the game will go through a 3rd step:

Step 3: Check if the experiment should end by throwing a **"virtual fair dice"**.

When the experiment reaches its final round, you will move to the final 4th step:

Step 4: Check if the threshold of the public account has been achieved and calculation of final payoffs

Step 3: Check if the game should end
The probability that the next round will happen after round 8 is 2/3. This means that at the end of each round, starting from round 8, a **"virtual fair dice"** with 6 faces, will be thrown. If the result is **"1"** or **"2"**, the game will end. Otherwise, when the result is "**3**", "**4**", "**5**" or "**6**", the game, will continue to the next round, in which the process is repeated. This means that the experiment will have a minimum of 8 rounds.

There is a **"virtual fair dice"** for each group. Thus, the experiment can have different rounds, depending on which group you are in.

You will be able to see on the screen the following text if the game continues: "*The result of the dice was X, which is different from "1" or "2". Therefore,* ***the experiment will continue*** *to the next round. Please, click now on the button* ***Ok****."*

However, if the result of the dice is "1" or "2", the screen will show the following text: "*The result of the dice was X. Therefore,* ***the experiment will end****. Please, click now on the button* ***Ok****."*

<u>Instructions the high uncertainty treatment (HU)</u>

The instructions for the treatment with high *timing uncertainty* differ from treatment 2 (low timing uncertainty) only in that the minimum number of rounds is 6 and the probability that the game ends afterwards is 1/5 (or 2/10). However, the average number of rounds remains 10. Below we describe only the sections that differ from the LU treatment:

General Information

The whole experiment consists **of minimum 6 and on average 10 rounds, but it could be more than that.**

The probability that the next round will happen after round 6 is 8/10. This means that at the end of each round, starting from round 6, a “virtual fair dice” with 10 faces, will be thrown. If the result is “**1**” or “**2**”, the game will end. Otherwise, when the result is “**3**”, “**4**”, “**5**”, “**6**”, “**7**”, “**8**”, “**9**” or “**10**”, the game will continue to the next round, in which the process is repeated.

Step 3: Check if the game should end

The probability that the next round will happen after round 6 is 8/10. This means that at the end of each round, starting from round 6, a **“virtual fair dice”** with 10 faces, will be thrown. If the result is “**1**” or “**2**”, the game will end. Otherwise, when the result is “**3**”, “**4**”, “**5**”, “**6**”, “**7**”, “**8**”, “**9**” or “**10**”, the game will continue to the next round, in which the process is repeated. This means that the experiment will have a minimum of 6 rounds.

Testing participant’s understanding

After reading the instruction, participants are requested to complete a short questionnaire that tests their understanding. Participants are not allowed to start the experiment until they answer to all questions correctly.

Experimental model and subject details

The results of our experiment were obtained by testing 246 participants (41% females) that were divided into 41 groups of six subjects each in a computerized experiment (using the software available at https://github.com/Socrats/beelbe (Fernández Domingos, 2020)). Most of the participants were bachelor/master/PhD students of either the Université Libre de Bruxelles or the Vrije Universiteit Brussel. The average age of participants was 24 (with a standard deviation of ~4 years). During each session of the experiments, participants were assigned randomly into groups and were not allowed to communicate (physical barriers were set up between them). Participants never knew who the other members of their group were.

In the control treatment (NU), 12 groups played the collective-risk dilemma (see main text) defined as in (Milinski et al., 2008) with the difference that the game was not framed as a climate change scenario, which makes the results more general to other scenarios of collective-risk where there is an uncertain deadline. Indeed, this type of N-person dilemma, combining non-linear and uncertain returns which are only reached in the future, are recurrent in many human collective endeavours, from public health measures to group hunting. In the treatment with low uncertainty (LU) and high uncertainty (HU), another 14 and 15 groups, respectively, played the variant of the game in which the final round was decided by a random process. After a minimum number of rounds (8 rounds in LU, and 6 in HU), the probability of the game ending after each round was w=1/3 and w=1/5 in LU and HU, respectively. To implement this uncertainty in LU (HU) a 6 (10) faces dice was thrown at the end of round 8 (6), and the game would continue if the result was higher than 2, thus generating the probability for ending the game in LU (HU).

Ethics

Ethical approval (reference ECHW_064) was obtained from the Ethical Commission for Human Sciences at the Vrije Universiteit Brussel to perform the experiments discussed in this manuscript.

Quantification and Statistical Analysis

In Figure 1A the averages and error bars (95% confidence interval) are computed across groups (n=12 for NU, n=14 for LU and n=15 for HU). For LU and HU, after the minimum number of rounds, m_0, n decreases, since some of the groups finished the game. Moreover, we only average values for groups which did not already achieve the target in the previous round. This also only happened in LU and HU. In LU, $n = 13$ for round 8, $n = 3$ for round 9, $n = 1$ for round 10. For HU, $n = 12$ for round 7, $n = 5$ for round 8, $n = 2$ for round 9, $n = 1$ for round 10. In Figure 1B each bar plot displays the proportion of groups that were successful for each of the treatments. 8 out of 12 groups were successful in NU, 9 out of 14 for LU and 7 out of 15 for HU. We performed a Chi-square test of independence ($P = 0.49952, n = 41, df = 2, \chi^2 = 1.38$) that issues that the differences between the fraction of successful groups among the treatments are not significant. In Figure 2 we calculate the fraction of successful players for each treatment that assume one of 3 contribution behaviours. The total number of players in each of the fractions ($C < F$, $C = F$, $C > F$) is (9, 21, 18) for NU, (13, 17, 24) for LU and (13, 8, 21) for HU. The error bars in Figure 3 were calculated as described in the "identifying conditional behaviour" section (see Transparent Methods for detail on the number of samples for the correlation analysis).

Identifying polarization of contributions

We divided the participants on our experiment based on their total contributions throughout the game and their relationship to what we call *fair donation* F. This value corresponds to the minimum donation required for a group to be successful, if all participants contribute the same, i.e., if all participants contribute exactly F the group will be successful. This value corresponds to half of the endowment ($F = E/2$). Therefore, we quantify the fraction of participants that contribute, in total, less ($C < F$), equal ($C = F$) or more ($C > F$) than F. In Figure 2, we show that the fraction of participants that contribute $C < F$ and $C > F$ grows with *timing uncertainty*, while *fair* players ($C = F$) diminish. We associate this divergence of donations to an increase of *polarized* reactions.

Identifying conditional behaviour

Conditional behaviours were assessed through the analysis of the average donation of each player as a function of the donations of the other group members in the previous round (see Figure 3). For this reason, the plot starts with the data after the first round. Also, we only take

into account the data of the experiment before the target is reached, i.e., when the public account contains less than 120 EMUs. We adopt a weighted linear regression so that samples with smaller errors were more important than those with large ones. The weight of a point i is calculated as $weight_i = \sigma_{\bar{x}}{}^{-1}$, where the errors $\sigma_{\bar{x}}$ were computed as :

$$\sigma_{\bar{x}} = \begin{cases} \infty, |x| = 1 \\ \sqrt{\frac{\sigma_{actions}}{|x|}}, |x| > 1 \end{cases}$$

Here $\sigma_{actions} = 4$, representing the range of the values an action can take, and $|x|$ indicates the number of samples used to calculate the average of the samples vector x. This way, points calculated from only 1 sample, almost do not count for the regression.

Polynomial fitting

Our analysis in Figure 3 of the main manuscript until focused on identifying linear correlations, and their sign, between participants' contributions and the contributions of their group mates. This choice allows us to extract meaningful relationships while avoiding overfitting. However, in some of the studied cases, as we would expect, the data dependency might be represented better by a curvilinear/polynomial model. Below we show that the conclusions presented in the main manuscript remain valid if high order fitting is chosen.

In Tables S5-S10, we display the results of an ANOVA test that compares polynomial regressions of different order (from 1 to 4) for all the 6 cases analysed in Figure 3 of the main manuscript. This test indicates whether increasing the order of the polynomial regression issues a significant improvement. In only 3 cases, a polynomial fit is significantly better than the linear model: no uncertainty and players meeting the target; low uncertainty and not meeting the target; and high uncertainty and not meeting the target. In Figure S5, we show results analogous to Figure 3 of the main manuscript, but using the model that fits the best each case. We show that our conclusions do not change, and perhaps, it is even clearer that, in the certainty case, when players meet the target, they adopt a slightly compensatory behaviour, while considerably lowering their contributions when the rest of the group adopt extreme actions: they donate either too much or too little.

Dependency analysis

The results in Table S2-S4 are all obtained using an ordered logistic regression or *cumulative link model* (Liu and Agresti, 2005), implemented in the *polr* function implemented in the MASS package in R. We estimate the probability of taking an action (0, 2 and 4) depending on a series of features. This allows us to study how the actions of the participants on the experiment depend on these features. We used this analysis to select the most relevant features for the behavioural representation of a participant, which was then used to produce the results of Figures 2 and 3 of the main manuscript. The *polr* function differs from a multimodal regression

in that it performs a ordered logistic regression, i.e., it takes into account the order of the labels. This is important in our case, since the contributions 0, 2 or 4 are ordered.

The features included in the regression are:

- public_account: The public account of the game, i.e., the cumulative sum of contributions of all participants
- private_account: The private account of the participant, i.e. the remaining endowment at a given round.
- round_donations_others: The donations of the members of the group in the previous round, without the focal player.
- actions_prev: The action of the focal player in the previous round.
- GameHalf2ndHalf: The half of the game in which the action takes place (1st half, 2nd half).
- rnd: A random binary number (0 or 1). This is used to check that the regression is producing correct results. The actions of the players should not depend on a random variable.
- public_account: GameHalf2ndHalf: Interaction term between the public account and the game half. Represents the degree to which there is an interaction between these two variables.
- actions_prev: GameHalf2ndHalf: Interaction term between the previous action of the player and the game half.
- private_account: GameHalf2ndHalf: Interaction term between the private account and the game half.

Clustering behaviours

Figure S4 shows a clustering analysis done over the behavioural data of participants in all three treatments. This analysis helps in identifying predominant behaviours in our data and motivates the strategies later chosen for our evolutionary model. In Figure S4A, the behaviour of each participant in the experiment (including all three treatments) is represented by a slope and an intercept. These parameters are obtained by regressing linearly the average contribution of each participant at a given round, $\overline{a_i}(t)$, in function of the total contribution of the other members of the group in the previous round, $a_{-i}(t)$. These points are then clustered using a DBSCAN algorithm (Birant and Kut, 2007), which identifies 4 clusters and a set of 29 outliers (with $eps = 0.18$[1]). The proportion of players of each treatment in the clusters is represented in the inset of Figure S4A. Figure S4B, C, F and G show the averaged contributions of players in each cluster in response to $a_{-i}(t)$. Players in cluster 0 (Figure S4B) display an almost unconditional response, and always contribute slightly above 2 EMUs. In contrast, players in cluster 1 (Figure

---

[1] This value was obtained by calculating the distance from each point to it closest neighbors, sorting the distances and finally calculating the point of maximum curvature.

S4C) are slightly compensatory, making higher contributions when the group fails to contribute enough. The players represented in these two clusters are more predominant in NU. Contrarily, players in cluster 2 (Figure S4F) belong mostly to LU and HU. This cluster exhibits a strong reciprocal response, with the average contribution of players being directly proportional to $a_{-i}(t)$. Players in cluster 3 (Figure S4G) belong in almost equal proportion to all 3 treatments, and they represent a strict fair behaviour, i.e., players contribute 2 EMUs unless the contributions of the group are too low. Finally, the set of 29 outliers is composed mostly of players of the LU and NU treatments and display opposing conditional behaviours (either compensatory – Figure S4D - or reciprocal – Figure S4E).

Game theoretical model

As an alternative to considering fully-rational agents, we describe the behavioural dynamics through an evolutionary process (Nowak, 2006; Perc et al., 2017, 2013; Sigmund, 2010; Traulsen and Hauert, 2009), in which individuals tend to copy those appearing to be more successful. More precisely, we analyse the behavioural dynamics in large (but finite) populations, when individuals revise their choice through imitation dynamics or social learning (Ewens, 2012; Fudenberg and Imhof, 2006; Traulsen et al., 2006).

We consider a finite population of *Z* individuals, who interact in groups of size *N*, in which they engage in the collective-risk dilemma with multiple rounds. Each individual can adopt one of the $n_s = 5$ strategies that mimic the behaviours observed on the experimental data: *always-2*, *always-4*, *always-0*, *compensator*, and *reciprocal*. The first three strategies are unconditional, i.e., they will always contribute the same, independently of the behaviour of the other group members. Differently, compensator and reciprocal are conditional strategies that adapt their behaviour to the rest of the group according to a threshold of total contributions per round. We consider this threshold to be 10, which is exactly half of the maximum contribution per round, without the focal player. In this manner, compensators always start contributing 2, and, afterwards, contribute 0 as long as the sum of contributions of the rest of group members in the previous round is above 10 units; otherwise they will contribute 4 EMUs. The behaviour of *reciprocal* is the exact opposite of *compensators*: They start contributing 2 EMUs and afterwards they contribute 4 EMUs as long the sum of contributions of the other members of the group in the previous rounds is above or equal to 10 units; otherwise they contribute 0. We do not assume any population structure, such that individuals are equally likely to interact with each other (the so-called well-mixed assumption). The success (or fitness) of an individual can be computed as the average payoff obtained from playing in multiple groups randomly sampled from the population. As a result, all individuals adopting one of the n_s=5 strategies can be seen as equivalent, on average.

To study the behaviour resulting from this set of strategies, we adopt a stochastic birth-death process combined with the pairwise comparison rule (Traulsen et al., 2006) to describe the

social learning dynamics of each of the strategies in a finite population. At each time-step, a randomly chosen individual A has the opportunity to revise their strategy by imitating (or not) the strategy of a randomly selected member of the population *B*. This update rule is known as the pairwise comparison rule (Traulsen et al., 2006). The imitation will occur with a probability which increases with the fitness difference between A and B. Here we adopt the Fermi function $p \equiv \left[1 + e^{\beta(f_A - f_B)}\right]^{-1}$, where β controls the intensity of selection (we use $\beta = 0.004$), and f_A (f_B) is the average fitness of A (B). In the limit of strong selection ($\beta \rightarrow \infty$), the probability *p* is either zero or one. In the limit of weak selection ($\beta \rightarrow 0$), *p* is always equal to $1/2$, irrespective of the fitness of *A* and *B*. We have checked that our results are qualitatively invariant for a broad range of values of β. In addition, we consider that, with a mutation probability μ, individuals adopt a randomly chosen strategy, freely exploring the strategy space.

Overall this adaptive process defines a large-scale Markov process, whose complete characterization becomes unfeasible as one increases the population size and number of strategies (Vasconcelos et al., 2017). However, this analysis of this stochastic dynamics is largely simplified in the limit of rare mutations. In this case, we are able to compute analytically the relative prevalence of each of the different strategies. Moreover, as shown in the Supplemental model results by means of large-scale computer simulations, this analytical approximation turns out to be valid for a much wider interval of mutation regimes. In this limit, when a new strategy appears through mutation, one of two outcomes occurs long before the occurrence of a new mutation: either the population faces the fixation of newly introduced strategy, or the mutant strategy is wiped out from the population. Hence, there will be a maximum of two strategies present simultaneously in the population (Fudenberg and Imhof, 2006; Imhof et al., 2005). This allows one to describe the behavioural dynamics in terms of a reduced Markov Chain of size $n_s = 5$, whose transitions are defined by the fixation probabilities ρ_{ij} of a single mutant with strategy j in a population of individuals adopting another strategy i. This probability is given by Equation (1) (Ewens, 2012; Karlin and Taylor, 1975; Traulsen et al., 2006):

$$\rho_{ij} = \left(1 + \sum_{m=1}^{N-1} \prod_{k=1}^{m} \frac{T^-(k)}{T^+(k)}\right)^{-1} \tag{1}$$

where $T^-(k)$ ($T^+(k)$) is the probability to decrease (increase) the number of individuals with the mutant strategy and can be obtained through Equation (2).

$$T^{\pm}(k) = \frac{k}{Z}\frac{Z-k}{Z-1}\left[1 + e^{\mp\beta[f_i - f_j]}\right]^{-1} \tag{2}$$

In the limit of neutral selection ($\beta = 0$), the fixation probabilities become independent of the fitness values and equal to 1/*Z*, offering a convenient reference scenario (see below). Since we

will have at most two different strategies in the population, we can calculate the fitness f_a of a strategy a, in a finite population of size Z and k individuals of strategy a and $Z-k$ of strategy b, as

$$f_a = \binom{Z-1}{N-1}^{-1} \sum_{k=0}^{N-1} \binom{k-1}{j} \binom{Z-k}{N-j-1} \Pi_{ab}(k+1) \tag{3}$$

where $\binom{Z-1}{N-1}^{-1} \sum_{k=0}^{N-1} \binom{k-1}{j} \binom{Z-k}{N-j-1}$ represents a hypergeometric sampling (sampling without replacement) of the population and $\Pi_{ab}(k+1)$ is the payoff of strategy a when facing strategy b, while the group is composed of $k+1$ individuals with strategy a (Santos and Pacheco, 2011). We numerically estimate pairwise payoffs Π_{ij} between every strategy pair i and j, for each possible composition of a group of N participants with k members of using strategy i and $N-k$ using strategy j. This is achieved by averaging over 10^3 games for each composition of the group and treatments (NU, LU and HU, using the same parameters adopted in the lab experiments).

The transition matrix $\Lambda = [\Lambda_{ij}]$ combines the different probabilities that a population in a homogeneous state S_i will end up in state S_j after the occurrence of one single mutation. This matrix is given by $\Lambda_{ij} = \frac{\rho_{ij}}{4} (j \neq i)$, whereas the diagonal of the transition matrix is defined by $\Lambda_{ii} = 1 - \frac{1}{4} \sum_{j \neq i} \rho_{ij}$ (note that 4 is the number of strategies minus one). The normalized left eigenvector associated with eigenvalue 1 of matrix Λ determines the stationary distribution p_w of the n_s-states Markov chain (Fudenberg and Imhof, 2006; Imhof et al., 2005; Karlin and Taylor, 1975). The stationary distribution characterizes the average time the population spends in each monomorphic state w.

To compute the expected fraction of successful groups, or group achievement (η), shown in Figure 4A, we weight the probability of success of each of the monomorphic states (populations with only one of the 5 strategies) by their predominance (given by the stationary distribution), i.e., $\eta = \sum_w p_w H_w$, where p is a row vector containing the stationary distribution, and H is a column vector containing the probability of success of each monomorphic state.

In our case, the probability of success is always 1 for populations of *always-2*, *reciprocal* and *always-4* players, while it is 0 for *always-0*. Compensators are only successful if the game lasts more than 10 rounds, therefore their probability of success is $(1-w)^{10-m_0}$, since the random process that decides the final round under *timing uncertainty* follows a geometric distribution. The calculation of the fraction of players that contribute less, equal or more than F, used in Figure 4B, is done in a similar fashion. In this case, we need to calculate the probability that a population consisting of each of the monomorphic states will contribute $C < F$, $C = F$, $C > F$. Since we can calculate the contributions of the players depending on the number of rounds of

the game, the computation of these probabilities is straightforward. The *always-2* players will always contribute F, *always-4* and *reciprocal* will contribute $C > F$, and *always-0* $C < F$. Once more, compensators will only contribute $C > F$ if the number of rounds is bigger than 10, otherwise they contribute $C < F$. We then multiply these probabilities by the stationary distribution to obtain the fraction of players that assume each of the previous behaviours. In Figure 4 all other parameters controlling the environment are set to be the same as in our lab experiments: the risk $r = 0.9$, the initial endowment $E = 40$ and the target is $\tau = 120$.

To obtain further intuition behind the emergence of cooperation, fraction of successful groups and strategies in each treatment, we also analyse the Markov Chain which defines the typical flow of probability between the different monomorphic states. Figure S6, shows the Markov Chain for two of the most paradigmatic scenarios: NU (Figure S6A) and HU (Figure S6B). In the Figure, arrows represent transitions favoured by natural selection, i.e., those whose fixation probability exceeds $1/Z$ (associated with the fixation probability of a mutant under neutral evolution). For instance, if an arrow goes from a state with strategy i to state with strategy j, it indicates that a mutant of strategy j will invade the population of strategy i with a probability which is higher than the one we obtain from neutral drift. The absence of an arrow indicates that such transition will occur with a low probability, i.e., with a probability lower than $1/\mathrm{Z}$. In this context, a strategy j is said to be evolutionary robust (ERS) (Nowak, 2006; Stewart and Plotkin, 2013; Traulsen and Hauert, 2009) if no mutant, adopting any other strategy, has a selective advantage. In other words, we can identify strategies that are evolutionary robust (a measure of stability) by noticing that there is no arrow emerging from its respective node.

Figure S6 illustrates a reference scenario in what concerns the invasion dynamics of strategies. In the absence of uncertainty, *always-0* and the *always-2* strategy are the only two evolutionary robust strategies. Moreover, given the number and strength of the transitions, the fair strategy can easily become the most prevalent behaviour. Differently, under high timing uncertainty, the *always-2* strategy starts to be invaded by *compensators* and *always-0* (Figure S6B), changing the ecology of behaviours observed in the absence of uncertainty. In fact, uncertainty can easily lead to complex behavioural dynamics, with cyclic dominances, and no evolutionary robust strategies, as illustrated in the right panel of Figure S6. *Reciprocators* can invade *always-0*, yet losing to compensators, which, in turn, are invaded by *always-0*. From this cyclic dynamic, even if not stable, both conditional strategies emerge as prevailing strategies, leading both to the emergence of reciprocity and polarization (see main text).

Supplemental model results

The limit of rare mutations allows us to conveniently employ a small-scale Markov chain to analytically compute the prevalence of each strategy. This is achieved by restricting the number of strategies simultaneously co-existing in a population (and groups) to a maximum of two. However, for arbitrary mutation rates, we may have a complex co-existence of more than two

strategies, calling for the adoption of large-scale computer simulations to confirm the validity of our theoretical results in other mutation (or exploration) regimes. To perform these computer simulations, we mimic the evolutionary process described above, with discrete steps involving imitation and mutation, yet without any constraint in the value of μ (here a free variable). At the beginning of each simulation, each individual randomly adopts one of the five strategies. In each generation, Z individuals are chosen to revise their strategy (in an asynchronous manner). For each combination of parameters, we run 30 simulations, each lasting 10^9 generations. The fitness of each individual A is calculated as the average return earned from 10^3 games played against $N-1$ individuals randomly selected from the population. The fraction of successful groups, or group achievement (η), is computed from the average fraction of groups that surpassed the threshold after a transient period of 10^5 generations. The same criterion is used to compute the overall level of polarization emerging from each simulation.

In Figure S7, we confirm that the stationary distribution obtained under the small mutation limit assumption is valid for a wide range of mutation values. Additionally, we also include the polarization results, considering only group combinations that achieve the target, with and without mutation (see Figure S8), showing that results discussed in the main text remain valid for a broad interval of exploration rates.

Supplemental results

In Figure 1 of the main text we display the averaged group contributions per round for each of the 3 treatments of our experiment. In Figure S1 we present the average cumulative contributions to the public account, i.e., the average content of the public account per round and per treatment. For each point, we only average the values for groups that have not achieved the target already in the previous round. There is an increase in earlier contributions for the treatments with uncertainty (LU and HU) with respect to the control (NU). By round 10, the target is achieved on average in all treatment. However, on the LU (low uncertainty) treatment, contributions already surpass 120 EMUs (the target) on average by round 8.

Moreover, Figure S2 shows the average contributions per round separated by whether groups reached or not the target. Here we observe that, for the successful groups (met target = True), the contributions in the treatments with uncertainty were always higher than in the control treatment (NU) before the minimum number of rounds. The figure also shows that for NU, the difference between successful groups and non-successful ones is mostly related to the contributions in the last two rounds. While the successful groups increase their contributions slightly by the end (compensate for other participants), the non-successful groups lower them. This highlights the importance of coordination when the time is certain. For the treatments with *timing uncertainty* this effect disappears, and successful groups contribute more and earlier.

In Figure S3 we show a comparison between the participants behaviours in the first and second parts of the game for the participants that met the target (this corresponds to Figure 2 of the main manuscript) and those that didn't. We can observe an increasing difference between the *generous* players (those that contribute $C > F/2$) in the first half of the game as *timing uncertainty* grows. This highlights the importance of early contributions to nudge participants into cooperation when there is a *shadow on the future*.

Table S1 shows the correlation results and the associated p-values for the results presented in Figure 3 of the main text. The correlations for the treatment without uncertainty are negative, which indicates a compensatory behaviour. For the treatments with uncertainty, however, these correlations are positive, indicating a reciprocal or Tit-for-Tat behaviour. Nevertheless, only the correlations for the successful players of LU and HU are statistically significant ($P < 0.001$).

4. Supplemental References

Birant, D., Kut, A., 2007. ST-DBSCAN: An algorithm for clustering spatial-temporal data. Data Knowl. Eng. 60, 208–221. https://doi.org/10.1016/j.datak.2006.01.013

Ewens, W.J., 2012. Mathematical population genetics., Second. ed. Springer Science+Business Media, LLC. https://doi.org/10.1007/978-0-387-21822-9

Fernández Domingos, E., 2020. beelbe: Web platform for behavioural experiments. GitHub Repos. https://doi.org/10.5281/zenodo.4000003

Fudenberg, D., Imhof, L.A., 2006. Imitation processes with small mutations. J. Econ. Theory 131, 251–262. https://doi.org/10.1016/j.jet.2005.04.006

Imhof, L.A., Fudenberg, D., Nowak, M.A., 2005. Evolutionary cycles of cooperation and defection. Proc. Natl. Acad. Sci. 102, 10797–10800.

Karlin, S., Taylor, H.M.A., 1975. A first course in stochastic processes., 2nd edn. ed. Academic Press, New York.

Liu, I., Agresti, A., 2005. The Analysis of Ordered Categorical Data: A.n Overview and a Survey of Recent Developments. Test 14, 1–73.

Milinski, M., Sommerfeld, R.D., Krambeck, H.-J., Reed, F.A., Marotzke, J., 2008. The collective-risk social dilemma and the prevention of simulated dangerous climate change. Proc. Natl. Acad. Sci. U. S. A. 105, 2291–2294. https://doi.org/10.1073/pnas.0709546105

Nowak, M.A., 2006. Evolutionary dynamics. Harvard University Press.

Perc, M., Gómez-Gardeñes, J., Szolnoki, A., Floría, L.M., Moreno, Y., 2013. Evolutionary dynamics of group interactions on structured populations: A review. J. R. Soc. Interface 10, 20120997. https://doi.org/10.1098/rsif.2012.0997

Perc, M., Jordan, J.J., Rand, D.G., Wang, Z., Boccaletti, S., Szolnoki, A., 2017. Statistical physics of human cooperation. Phys. Rep. 687, 1–51. https://doi.org/10.1016/j.physrep.2017.05.004

Santos, F.C., Pacheco, J.M., 2011. Risk of collective failure provides an escape from the tragedy of the commons. Proc. Natl. Acad. Sci. U. S. A. 108, 10421–10425. https://doi.org/10.1073/pnas.1015648108

Sigmund, K., 2010. The Calculus of Selfishness. Princeton University Press. https://doi.org/10.1038/4641280a

Stewart, A.J., Plotkin, J.B., 2013. From extortion to generosity, evolution in the Iterated

Prisoner's Dilemma. Proc. Natl. Acad. Sci. U. S. A. 110, 15348–15353. https://doi.org/10.1073/pnas.1306246110

Traulsen, A., Hauert, C., 2009. Stochastic evolutionary game dynamics. Rev. Nonlinear Dyn. Complex. 2, 28–61.

Traulsen, A., Nowak, M.A., Pacheco, J.M., 2006. Stochastic dynamics of invasion and fixation. Phys. Rev. E - Stat. Nonlinear, Soft Matter Phys. 74, 011909. https://doi.org/10.1103/PhysRevE.74.011909

Vasconcelos, V. V., Santos, F.P., Santos, F.C., Pacheco, J.M., 2017. Stochastic Dynamics through Hierarchically Embedded Markov Chains. Phys. Rev. Lett. 118, 058301. https://doi.org/10.1103/PhysRevLett.118.058301
